# Yoga Nidra for hypertension: A systematic review and meta-analysis

**DOI:** 10.1016/j.jaim.2023.100882

**Published:** 2024-03-13

**Authors:** Navdeep Ahuja, Praag Bhardwaj, Monika Pathania, Dilasha Sethi, Arjun Kumar, Ashwin Parchani, Akshita Chandel, Aashish Phadke

**Affiliations:** a Dept. of Medicine, All India Institute of Medical Sciences, Rishikesh, Uttarakhand, India; b Division of Yoga and Life Sciences, Swami Vivekananda Yoga Anusandhana Samsthana - SVYASA, Bangaluru, Karnataka, India; c Dept. of General Medicine, All India Institute of Medical Sciences, Bilaspur, Himachal Pradesh, India; d Division of Endocrine and Metabolic Disorders - Lifestyle Modulations and Yoga Modules, Kasturba Health Society - Medical Research Centre, Mumbai, Maharashtra, India

**Keywords:** CVD risk, Cardiac health, Mind body medicine, Mindfulness, Non-communicable disease, Stress management

## Abstract

**Background:**

Hypertension is a prevalent chronic condition that affects a substantial proportion of the world's population. Medications are commonly prescribed for hypertension management, but non-pharmacological interventions like yoga are gaining popularity.

**Objective:**

The purpose of this systematic review and meta-analysis is to assess the efficacy of Yoga Nidra (YN) for the management of hypertension.

**Methods:**

A systematic review and meta-analysis of clinical trials, i.e., non-randomized and randomized controlled trials (RCTs) was conducted to investigate the effects of YN on hypertension. PubMed, the Cochrane Library, SCOPUS, and EBSCO were searched for relevant studies published up to September 2022. The quality of the included studies was assessed using the Cochrane risk of bias tool. The primary outcome measure was the change in systolic blood pressure (SBP) and diastolic blood pressure (DBP) after YN intervention, analyzed as weighted mean difference (WMD), in comparison to control groups. The random-effects model was used for the meta-analysis. Risk of bias was assessed for RCTs and non-RCTs using Cochrane's RoB-2 and ROBINS-I tools, respectively.

**Results:**

Five RCTs and three Non-RCTs involving a total of 482 participants (239 for YN vs 243 for controls) were included in this review. The meta-analysis indicated that YN significantly reduced SBP (WMD = 12.03 mm Hg, 95% CI [7.12, 16.93], *Z* = 4.80, *p* < 0.00001) and DBP (WMD = 6.32 mm Hg, 95% CI [3.53, 9.12], *Z* = 4.43, *p* < 0.00001) compared to control groups. The overall risk of bias for the three RCTs was high, whereas for the five non-RCTs, one had an overall moderate risk while the other four had an overall serious risk of bias.

**Discussion:**

This systematic review and meta-analysis provides evidence supporting the efficacy of YN as a complementary therapy for hypertension management. YN is a safe, cost-effective, and easily accessible intervention that primarily relies on interoception and induces a deep relaxation response in practitioners, aiding them in coping with various components of high blood pressure, such as stress, vascular inflammation, peripheral vascular resistance, etc. Our understanding of the mechanisms of YN is constantly evolving, and there is a need for further research to fully explore and appreciate the significance of this ancient science and its potential efficacy on BP. Considering the results and the multifactorial role of YN, it can act as a safe and reliable adjuvant therapy to complement the pharmacological treatment of hypertension. However, further studies with larger sample sizes, longer follow-up periods, and homogenous populations are warranted.

**Conclusion:**

This meta-analysis suggests that YN is effective in reducing SBP and DBP, particularly in individuals with hypertension. The results highlight the potential of YN as a complementary therapy for hypertension management. Healthcare providers may consider recommending YN to patients with hypertension as an adjuvant therapy to medication. Further studies are required to identify standardized optimal forms and durations of YN best suited for hypertension management.

## 1 Introduction

Hypertension, also known as high blood pressure (BP), is defined by the American Heart Association (AHA) as a chronic medical condition characterized by consistently elevated BP, with systolic blood pressure (SBP) of more than 130 mm Hg or diastolic blood pressure (DBP) of more than 80 mm Hg [1]. This increased pressure puts significant strain on the heart and blood vessels, raising the possibility of serious complications, affecting over a billion people worldwide [2]. In India, the overall prevalence of hypertension is 29.8%, with 27.6% in rural populations and 33.8% in urban [3]. Hypertension accounts for approximately 54% of all strokes and 47% of coronary artery diseases, thus causing more deaths than any other cardiovascular risk factors [4]. The global burden of disease study ranks elevated SBP as the leading metabolic risk factor attributable to disability adjusted life years [5], while the overall number of cardiovascular deaths continues to rise as societies age [6].

There are two broad categories of hypertension: essential hypertension, which affects 85% of all patients without an identifiable cause; and secondary hypertension, which is caused by underlying conditions such as renal artery stenosis, pheochromocytoma, adrenal adenoma or single gene mutations. Investigating the pathophysiology of hypertension is still a work in progress as several mechanisms, including increased sympathetic drive, renin-angiotensin-aldosterone system activation, receptor-mediated vasoconstriction or impaired vasodilation, increased reactive oxygen species, genetic influences, and several other immune-mediated mechanisms, all work together to contribute to hypertension [7,8]. Understanding these mechanisms has aided in the development of hypertension-specific therapies.

As per the AHA, management of hypertension in all its stages involves pharmacological and non-pharmacological interventions [1]. Weight loss, low sodium intake, physical activity, and moderation in alcohol consumption are the main non pharmacological interventions used in the management of hypertension [1]. Pharmacological interventions include categories of drugs like calcium channel blockers, angiotensin converting enzyme inhibitors, angiotensin receptor blockers, beta blockers, diuretics and sympatholytic drugs. Anti-hypertensive medications have a crucial role in hypertensive emergencies and urgencies. Despite their proven efficacy, high drug costs and drug interactions pose a significant barrier in patient's adherence to medication. Long-term use of certain drugs can result in disabling side effects such as nausea, muscle cramps, dizziness, dry mouth, and so on [9,10]. Moreover, long-term use of antihypertensive medication has been linked to a lower quality of life and financial constraints [12]. This adds to mental stress and poor mental health of the hypertensive patient [11]. Discontinuation of the prescribed drug reverts the patient's pathophysiology, resulting in high BP and contributing to high rates of poorly controlled hypertension [10]. Therefore, in chronic management of hypertension, antihypertensive drugs can work in unison with non-pharmacological interventions for a better patient outcome. Thus, lifestyle changes should be used to prevent or treat hypertension, with or without medication. Although dietary changes and exercise are beneficial, they rarely manage hypertension on a long-term basis due to low patient motivation and effort [13]. Given the shortcomings of currently available strategies, it is prudent to investigate newer, more cost-effective, and easier-to-administer therapies to combat the ever-growing morbidity of hypertension that may convey the benefits of long-term adherence [8].

Yoga is a complementary and alternative therapeutic modality that originated in India and consists of physical postures, breathing practices, relaxation exercises and meditation techniques [14]. Yoga practice is suggested to lower BP by lowering stress, increasing parasympathetic activation and changing baroreceptor sensitivity [14,15]. Previous research suggests that yoga is an effective adjunct therapy for hypertension management. The AHA scientific statement from 2017 states that meditation techniques like the Transcendental Meditation can possibly benefit on cardiovascular risk and can be considered for treating individuals with BP above normal thresholds [16]. However, due to the wide variety of yoga practices and the variable quality of the research, it is difficult to recommend any specific yoga practices [15].

Amongst the multitude of yoga techniques, Yoga Nidra (YN) is a supine relaxation technique performed in Shavasana (corpse pose) [17,18] and is sometimes referred to as “yogic sleep” [18,19]. It appears similar to hypnosis, but YN differs to the effect that the practitioner retains his free will and is mentally aware of his surroundings and himself [18,19]. Yoga masters describe the mental state in YN as a relaxed yet fully aware state of consciousness, in which the mind is neither asleep nor awake [19] and has been equated to the hypnagogic state of consciousness i.e., the transitioning phase from wakefulness to sleep [17]. The Ashtanga Yoga of Maharishi Patanjali advocates “Pratyahara,” or withdrawing the mind and mental awareness from the outwardly manifested sensory channels. Pratyahara is the primary modality used by YN to internalize awareness and induce interoception [17]. YN practices also use techniques such as body/breath awareness, progressive muscular relaxation, and guided imagery to lead the practitioner's mind through a specific series of mental images and sensations, distinguishing it from other relaxation techniques such as meditation or mindfulness [17,18]. All of this work together to activate the relaxation response in the body which results in autonomic changes and BP regulation. YN has been shown to have a variety of health benefits including stress and anxiety reduction, improved sleep and overall well-being, and is quickly becoming popular among the general public due to its broad applicability, safety, and feasibility [18,20]. Growing research backs the use of YN in the management of hypertension, and there is ever-expanding evidence in favor of YN [20].

Despite numerous investigational studies, there is no conclusive pooled evidence on the effect of YN on hypertension and BP. This study aims to conduct a comprehensive literature review to qualitatively and quantitatively synthesize the anti-hypertensive effects of YN among studies assessing healthy and diseased adults, with mean BP thresholds exceeding normal values (SBP >120 mm of Hg and DBP >80 mm of Hg). This pooled effect may help determine whether YN can be included as an evidence-based adjunct therapy for hypertensive patients that is low-cost, feasible, and easy to implement with minimal contraindications.

## 2 Methods

### 2.1 Search strategy and sources

A systematic literature search was performed in the electronic databases of PubMed, Scopus, Cochrane Library, and EBSCO Essentials for eligible studies published until September 20, 2022. Relevant keywords, including hypertension, blood pressure, prehypertension, mean arterial pressure, systolic blood pressure, diastolic blood pressure, SBP, DBP, elevated blood pressure, yoga nidra, yoganidra, yoga-nidra, shavasana, psychic sleep, corpse pose, yogic relaxation, yogic sleep, yogic rest, supine yoga relaxation, and guided sleep meditation, were used for searching literature using Boolean operators “AND” and “OR”. All searches were restricted to titles, abstracts and keywords of the respective databases and limited to English language only. Searches were re-run by the authors before analysis to check for any discrepancies.

### 2.2 Eligibility criteria

Only experimental and quasi-experimental studies i.e., randomized controlled trials (RCTs), non-RCTs and clinical trials having at least two comparable population groups were selected based on their eligibility for the following criteria framed in PICO format (Population Intervention Comparator Outcome).

#### 2.2.1 Inclusion criteria

· Population: Studies among adults (>18 years) belonging to either gender (male/female) and having any of the following: Elevated BP (SBP = 120–129 mm of Hg or DBP <80 mm of Hg); Stage 1 Hypertension (SBP = 130–139 mm of hg or DBP = 80–89 mm of Hg); Stage 2 Hypertension (SBP ≥140 mm of Hg or DBP ≥90 mm of Hg); Hypertension Crisis (SBP >180 mm of Hg or DBP >120 mm of Hg) [21].
· Intervention: Studies utilizing YN alone or incorporating YN as a major part of yoga intervention i.e., having at least 50% of the time devoted to YN out of the total intervention. YN was defined as “Supine relaxation or Shavasana involving awareness of breath, rotational awareness of consciousness, or an act of visualization”.
· Comparator: Studies comparing YN with any active or passive interventions, standard care, waitlist groups and no intervention.
· Outcome: Studies assessing change in SBP and DBP.
· Time: Studies with YN interventions of any duration and using any mode of delivery i.e., online, offline, in-group, in-person etc., published before September 2022.

#### 2.2.2 Exclusion criteria

· Studies with participants <18 years of age or participants with normal BP (SBP <120 mm Hg and DBP <80 mm Hg).
· Studies utilizing interventions other than YN or not meeting the intervention criteria stated above.
· Studies not assessing change in SBP and DBP.
· Single arm trials, proxy studies, qualitative studies, observational studies, research protocols, unpublished thesis or dissertations, abstracts and letters to editors.
· Studies in languages other than English.

### 2.3 Selection process

Search results from different databases were compiled in a single excel file and checked for duplicates using Rayyan, an internet-based application for systematic reviews [22]. After removing duplicates, two co-authors independently screened the remaining studies for potential reports worth inclusion using a screening checklist form created on the online platform “Google Sheets” to be filled out simultaneously. Prior to selection, each study's abstract was thoroughly assessed to ensure its eligibility based on the PICO format of the original review question. During the screening procedure, any conflicts that arose were discussed with a third co-author and resolved by consensus. After the screening process was complete, the authors double-checked every selected study for any errors.

### 2.4 Data extraction

Three review authors independently extracted data from studies on basic characteristics (e.g., author, year, country, study design, etc.), participant details (e.g., demographics, age, gender, comorbidities, etc.), intervention details (e.g., allocation ratio, nature, length, and duration of intervention, dropouts, etc.), outcome data and analysis (assessment time points, pre and post intervention results, statistical tests used, etc.) and other details (e.g., protocol registration, participant's informed consent, ethical clearance, conflict of interest, etc.). Data extracted on the above points was recorded using google sheets, based on the data collection form for intervention reviews (RCTs and non-RCTs) by Cochrane.

### 2.5 Risk of Bias Assessment

Included studies were assessed for risk of bias using the Revised Cochrane Risk-of-Bias Tool for Randomized Trials (ROB-2) and Risk-of-Bias in Non-Randomized Studies of Interventions (ROBINS-I) tool, as per their respective study designs. Assessments were performed independently by three co-authors and judgments regarding the risk of bias were made after consensus and under scrutiny of a senior co-author.

### 2.6 Quantitative assessment

Cochrane's RevMan software (Version 5.4) was used to conduct a meta-analysis and generate the forest plot. For statistical analysis, the mean differences and standard deviations of the differences between the intervention groups and the control groups were sought from the data provided within the selected manuscripts. The calculations were performed using the Cochrane handbook's provided formulas. The I^2^ statistic was used to test for heterogeneity, with I^2^ values above 50% indicating significant heterogeneity. Tau^2^ (τ^2^) was used to determine the proportion of heterogeneity explained by subgroup differences.

### 2.7 Protocol registration

The review protocol was registered prospectively with the International Prospective Register of Systematic Reviews (PROSPERO) on January 02, 2022, under registration number CRD42022283667.

## 3 Results

### 3.1 Search results

**Fig. 1:**
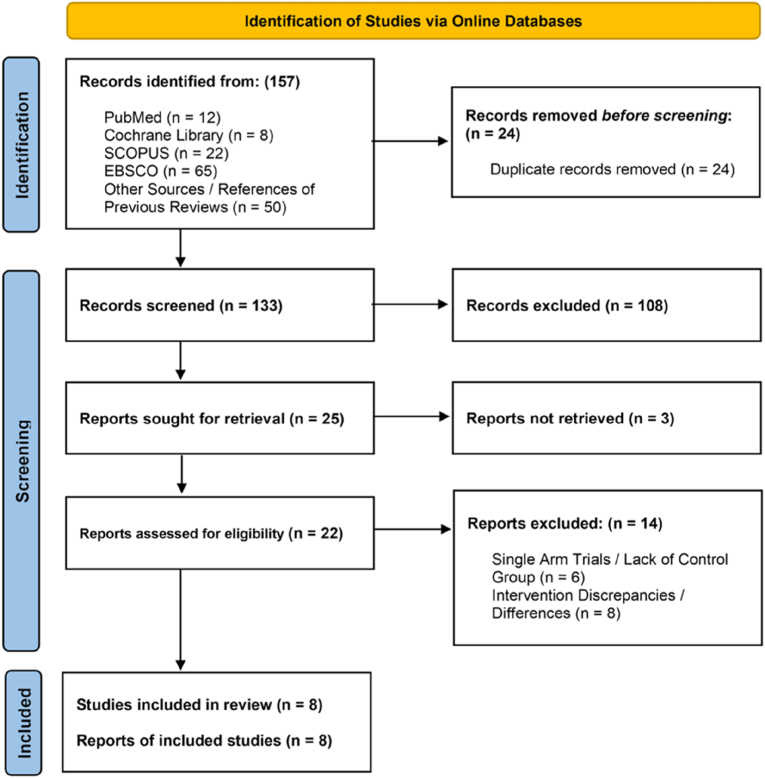
Prisma flow diagram.

The searches conducted in the electronic databases yielded a total of 157 results. The PRISMA (Preferred Reporting of Systematic Reviews and Meta-Analyses) flowchart in Fig. 1 depicts the number of search results returned by each database. Along with online searches, abstracts of 50 relevant references screened from seven previous reviews were also checked for inclusion [[23], [24], [25], [26], [27], [28], [29]]. There were 24 duplicate studies among the search results, which were eliminated before the screening process. According to inclusion and exclusion criteria, 108 of the 133 remaining studies were excluded during hand screening by co-authors based on information in the titles and abstracts. From the 25 studies considered for in-depth analysis, full papers were retrieved for 22 of the studies. 14 studies [[30], [31], [32], [33], [34], [35], [36], [37], [38], [39], [40], [41], [42], [43]] were excluded during the inclusion evaluation for the following reasons: single-arm trials [30,31], lacking comparative control groups [[30], [31], [32], [33]], variations in outcome measurement [34], not meeting the required baseline BP thresholds [35], not meeting the YN intervention criteria [[36], [37], [38], [39], [40], [41], [42], [43]], having supplementary interventions that reduced the proportion of YN (<50% of total intervention duration) [[36], [37], [38], [39], [40], [41]], lacking details on the length of YN [42,43]. A total of eight studies i.e., three RCTs [44,46,48] and five non-RCTs [45,47,[49], [50], [51]] were included for systematic review and meta-analysis.

### 3.2 Study characteristics

The sample sizes for the studies ranged from 30 to 150. The mean age of participants in most of the studies was between 40 and 60 years old, except for one study that had a mean age of under 30 years in each group [44]. Thangam et al. [51] enrolled elderly participants with both stage 1 and 2 hypertension, Anjana et al. [46] included stage 1 hypertensives, while Patel et al. [48,50], Devraj et al. [49] and Deepa et al. [47] included Stage 2 hypertensives. Participants without hypertension were included in the two studies by Monika et al. [44] and Lijun Li et al. [45], which focused on females with menstrual irregularities and patients undergoing colonoscopy, respectively. However, the participants in both studies had elevated baseline BP (SBP >120 or DBP >80 mm of Hg), so both studies were taken into consideration for our review. Three studies had patients who were on anti-hypertensive drugs [[46], [47], [48]], two studies included patients of which some were on antihypertensive agents [49,50], two studies did not mention the use of antihypertensive agents [45,51], and one study had subjects with elevated BP, not on any medication [44]. The duration of YN intervention ranged from 15 to 40 min except for one study that did not define the duration [50]. Two of the included articles assessed the effects of YN along with standard pharmacological medications versus standard medication alone [44,51]. Two studies by Patel et al. [48,50] have used psychophysical relaxation based on yogic principles, reinforced by biofeedback instruments, along with pharmacological medications. Their intervention meets our defined criteria for YN, and compares for self-relaxation and standard medications among age and gender matched controls. The biofeedback instrument provided patients with continuous feedback about their SNS activity through an auditory signal, allowing for deeper relaxation while their BP levels were monitored throughout the yogic relaxation sessions. Four studies used other co-interventions in addition to YN, such as Om chanting [46,47], a brisk walk [47,49], joints rotation [47], and music therapy [45]. However, YN accounted for more than 50% of the time in each study's experimental group intervention, making it a crucial behavioral component of the overall intervention. The duration of follow-up ranged from 15 days to 12 months, with an average of three months in most studies. Table 1 details the characteristics of individual studies.

**Table 1:** Characteristics of included studies

**Author, Year and Country**	**Study Design**	**Randomization/Matching, Allocation Concealment and Blinding Procedures**	**Population Characteristics and Sample Size**	**Experimental Group Sample Characteristics**	**Control Group Sample Characteristics**	**Intervention Details for Experimental Group**	**Intervention and Details for Control Group**	**Intervention and Follow up Duration**
Monika et al., 2012, India [44]	RCT	Block randomization of four. Random number generator utilized for SNOSE based allocation. Blinding procedures were not mentioned.	Females with menstrual irregularities (on medications). *N* = 150. with mean BP Elevated at baseline (SBP >120 mm of Hg).	75 enrolled, 65 completed the trial. Mean age: 28.53 years.	75 enrolled, 61 completed the trial. Mean age: 27.62 years.	YN for 35–40 min (guided by a trained yoga instructor) along with standard medications.	Standard medications alone.	Intervention duration was for five days in a week for six months for both the groups.
Lijun Li et al., 2018, China [45]	Non-RCT.	No details about matching of controls. Blinding procedures were not mentioned.	Adults over 18 years of age undergoing colonoscopy; *N* = 11 with mean BP Elevated at baseline (SBP >120 and DBP >80 mm of Hg).	50 enrolled, 40 completed the trial. Mean age: 47 years. Gender: 60% M, 40% F	Ctrl group with music: 46 enrolled, 35 completed the trial; Mean age: 47.07 years; Gender: 54.35% M, 45.65% F. Ctrl group with self-relaxation: 48 enrolled, 38 completed the trial; Mean age: 48.96 years. Gender: 45.83% M, 54.17% F.	YN for 38 min during colonoscopy using a Audio recording played using headphones.	Ctrl group (music): Listening to instrumental music during Colonoscopy. Ctrl group (self-relaxation): Attempt self-relaxation during Colonoscopy.	Intervention for all the three groups was implemented 10 min before scope insertion (at baseline) and lasted until the scope was completely removed.
K. Anjana R, 2022, India [46]	RCT.	Block randomization method. Allocation concealed using SNOSE. Blinding procedures were not mentioned.	Subjects diagnosed with HTN (Stage-1), undergoing HTN specific diet and pharmacological therapy. *N* = 65.	*n* = 34. Mean age: 49.13 years. Gender: 15 M, 19 F.	*n* = 31. Mean age: 43.90 years. Gender: 14 M, 17 F.	Five minutes of Om chanting and 20 min YN guided by an instructor.	No intervention.	Intervention duration was for two months for both the groups.
Deepa et al., 2012, India [47]	Non-RCT.	Matched case control design. Blinding procedures were not mentioned.	Subjects diagnosed with mild to moderate essential HTN (Stage-2) on anti-hypertensive drug therapy. N = 30	*n* = 15. Mean age: 54.33 years. Gender: eight M, seven F.	*n* = 15. Mean age: 53 years. Gender: eight M, seven F.	Vajrasana, Pranayama for 5 min in Sukhasana followed by “Om” Meditation for 5 min in Shavasana and YN for 45 min guided by a trained yoga master. Sessions done at least five days a week.	No intervention.	Intervention duration was for 12 weeks for both the groups.
Patel, 1975, England [48]	RCT with one way crossover design.	Age and gender matched controls. Sequential consecutive random sampling. Outcome assessor was blinded.	Hypertensive adults (Stage-2) on pharmacological treatment for at least six months. *N* = 34.	*n* = 17. Mean age: 59.5 years. Gender: six M, 11 F.	*n* = 17. Mean age: 58.6 years. Gender: seven M, 10 F.	35-min sessions of Biofeedback assisted yogic relaxation provided manually.	Placebo therapy (general relaxation) without any instructions and without any biofeedback instruments.	Both the groups went through 12 sessions, twice a week for six weeks.
Devraj et al., 2021, India [49]	Non-RCT.	Blinding procedures were not mentioned.	Subjects with HTN (Stage-2), both with and without medication. *N* = 74.	*n* = 31. Mean age: 54.61 years. Gender: 24 M, seven F.	*n* = 43. Mean age: 49.645 years. Gender: 30 M, 13 F.	Warm-up exercises like body rotation and joint rotations after 2–3 min of brisk walk followed by 35 min of YN daily using audio CD player, under supervision of a Yoga instructor. Sessions done at least five days a week.	No intervention.	Intervention duration was for twelve weeks for both the groups.
Patel, 1975, England [50]	Non-RCT.	Age and gender matched controls. Blinding procedures were not mentioned.	Hypertensive patients (Stage-2) mostly on anti-hypertensive medications. *N* = 40.	*n* = 20. Mean age: 57.35 years. Gender: nine M, 11 F.	*n* = 20. Mean age: 57.2 years. Gender: nine M, 11 F.	Standard medications along with 30 min of guided Yogic Relaxation Sessions provided manually, aided with biofeedback instruments, were given thrice a week.	Standard medications along with self-relaxation on the couch without any instructions and without any biofeedback instruments.	Interventions lasted for three months for both the groups. After three months, patients were encouraged to continue self-practice for nine months of follow up.
Thangam FE and Bharathi D, 2016, India [51]	Non-RCT.	Blinding procedures were not mentioned.	Elderly patients (>60 years) at old age homes having Diabetes, HTN (Stage-1,2) and Arthritis. *N* = 35	*n* = 20. Gender: six M, 14 F.	*n* = 15. Gender: two M, 13 F.	20 min of YN once daily (method of administration not reported) along with standard care.	Standard drug care alone.	Intervention duration was for 15 days for both the groups.

**RCT:** Randomized Controlled Trial; **SNOSE:** Sequentially Numbered Opaque Sealed Envelopes; **Ctrl:** Control; **Exp:** Experimental; **YN:** Yoga Nidra; **HTN:** Hypertension; **F:** Females; **M:** Males.

### 3.3 Risk of Bias Assessment

The following sections elaborate on the Risk of Bias for the three RCTs and five non-RCTs, assessed using Cochrane's ROB-2 and ROBINS-I tools, respectively.

#### 3.3.1 Assessments for RCTs using ROB-2

**Fig. 2:**
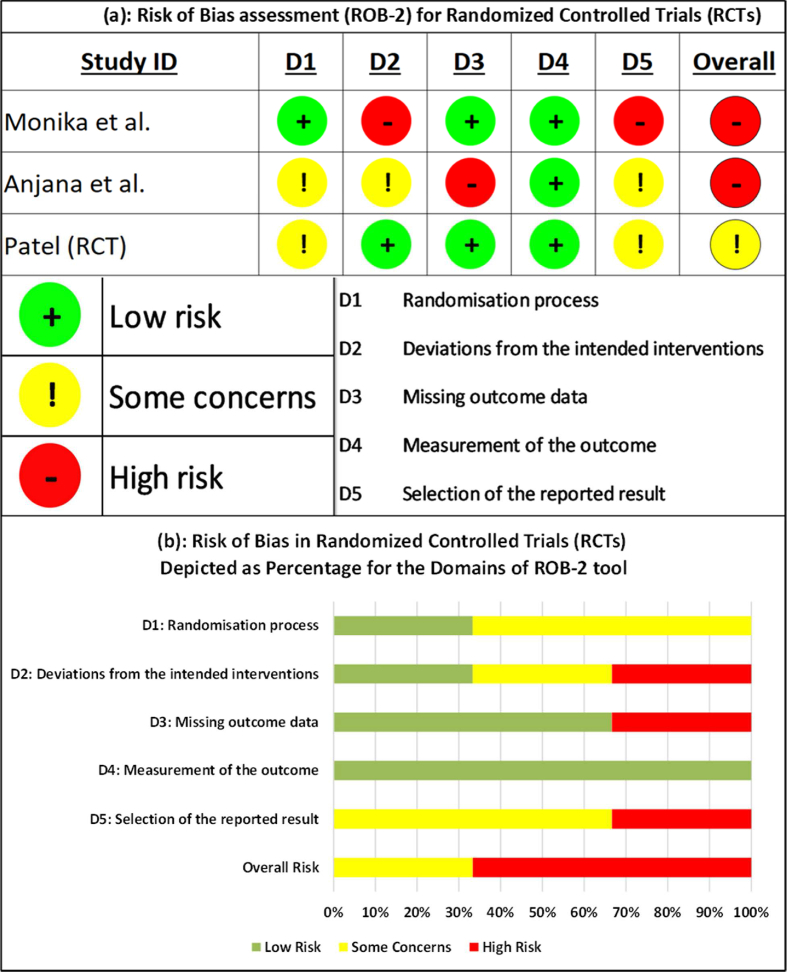
(a) Risk of bias assessment for randomized controlled trials (RCTs). (b) Risk of bias assessment for Randomized Controlled Trials (RCTs) depicted as percentage for the domains of ROB-2 tool.

For the first domain of bias due to the randomization, two of the three RCTs had some concerns as there were observable differences in outcomes at baseline [46] and a lack of clarity about the method of randomization [48]. Under domain two, i.e., bias due to deviations from intended interventions, one study had high risk due to a significant number of people dropping out with no specific reasons mentioned [44], while another study had some concerns due to a lack of intention to treat analysis [46]. For domain three of bias due to missing outcome data, one study had high risk due to non-disclosure of outcome data of drop-outs and no proper justification for the drop-outs [46]. There was a low risk for the fourth domain, i.e., bias due to measurement of outcomes, since all the three included studies utilized BP and other objective measures, measured at parallel time points for both experimental and control groups. For the fifth domain of bias due to selective reporting of the results, some concerns were observed for two studies due to the lack of a pre-trial protocol and statistical analysis plan [46,48], while high risk was observed for one study for reporting comparisons between outcomes measured at different time-points [44]. All of the above add up to a high overall risk of bias for the cumulative results of all three randomized controlled trials taken together. Fig. 2(a) depicts the risk of bias assessed for individual RCTs, while Fig. 2(b) depicts the percentage of risk of bias assessed for different domains of ROB-2 tool.

#### 3.3.2 Assessments for non-RCTs using ROBINS-I

**Fig. 3:**
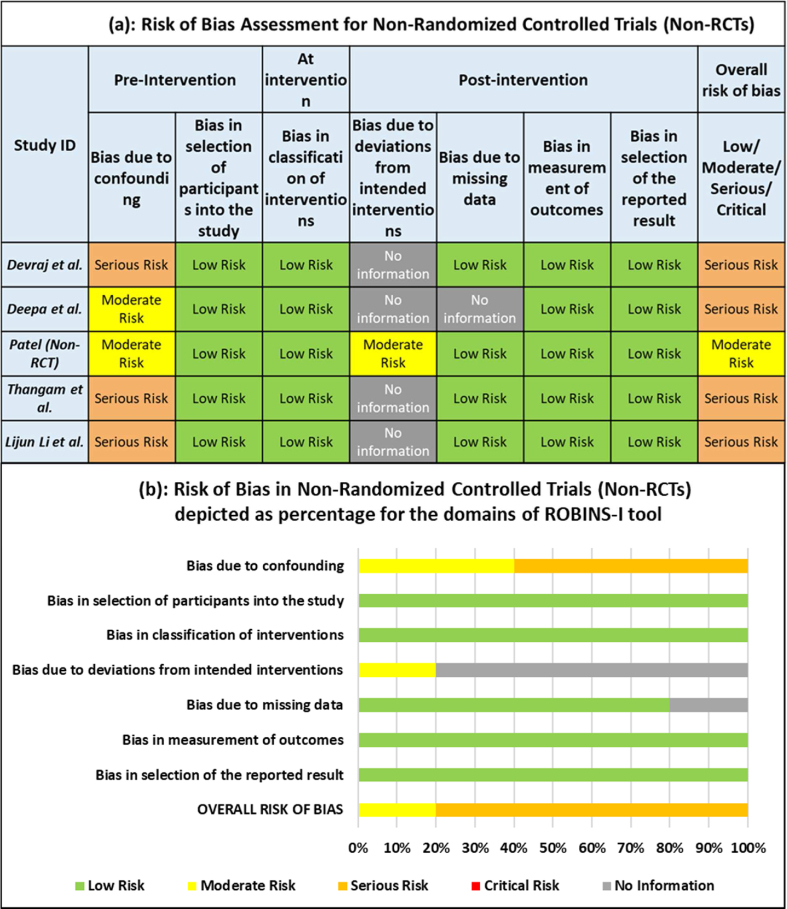
(a) Risk of bias assessment for non-randomized controlled trials (Non-RCTs). (b) Risk of bias assessment for Non-Randomized Controlled Trials (Non-RCTs) depicted as percentage for the domains of ROBINS-I tool.

Out of the five non-RCTs, one had an overall moderate risk [48], rest all four had an overall serious risk of bias [45,47,49,51]. Since hypertension can be influenced by confounding factors such as age, gender, diet, lifestyle, comorbidities, etc. the bias due to initial confounding is moderate for two studies [47,50] and serious for three studies [45,49,51] mostly due to a lack of adequately matched controls. Under the domain of bias due to deviation from intended intervention, four studies had no information available on prior protocols and statistical analysis plans [45,47,49,51], while the fifth study [50] had moderate risk. Except for one study which had no information on the missing outcome data [47], all the five studies had a low risk for the remaining five domains of bias i.e., selection of participants, classifications of interventions, missing data, measurements of outcomes and selective reporting of results. Fig. 3(a) depicts the risk of bias assessed for individual non-RCTs while Fig. 3(b) depicts the percentage of risk of bias assessed for different domains of ROBINS-I.

### 3.4 Meta-analysis

In three studies [44,45,47], the standard deviations of the difference in BP were not provided. Hence, the imputation method described in the Cochrane document was used [52]. One of the studies [48] was chosen to calculate the correlation coefficient because this study provided both the difference in means as well as the standard deviation between pre- and post-intervention in both the experimental and control groups and had a relatively consistent correlation value for all the outcomes. The overall effect (*Z*) favors YN as an intervention over the control, with the direction of the effect, for both SBP and DBP, favoring YN (overall effect for SBP: *Z* = 4.80 [*p* = 0.00001]; overall effect for DBP: *Z* = 4.43 [*p* = 0.00001]). The heterogeneity is very high for both SBP (*τ*^*2*^ = 39.08; *χ*^*2*^ = 95.81, *df* = 7 [*p* < 0.00001]; *I*^*2*^ = 93%) and DBP (*τ*^*2*^ = 12.88; *χ*^*2*^ = 67.82, *df* = 7 [*p* < 0.00001]; *I*^*2*^ = 90%). Table 2(a), Table 2(b)(a) and 2(b) show the statistical computations of the meta-analysis of all the included RCTs and non-RCTs, for both SBP and DBP, respectively. Fig. 4(a), Fig. 4(b)(a) and (b) depict the forest plots of all the RCTs and non-RCTs for both SBP and DBP, respectively.

**Table 2(a):** Statistical Computations of the Meta-analysis of all the included RCTs and non-RCTs for the outcome of Systolic Blood Pressure

**Study ID**	**Yoga Nidra**			**Control**			**Weight**	**Mean Difference**
**(First Author)**	**Mean**	**SD**	**Sample Size (*****n*****)**	**Mean**	**SD**	**Sample Size (*****n*****)**	**in %**	**IV, Random, 95% CI**
**Anjana et al.**	7.6	3.58	31	0.88	1.01	34	15.9%	6.72 [5.41, 8.03]
**Deepa et al.**	22.13	11.08	15	11.06	11.18	15	11.3%	11.07 [3.10, 19.04]
**Devraj et al.**	24.22	22.87	31	3.58	10.39	43	10.7%	20.64 [12.01, 29.27]
**Lijun Li et al.**	0.72	13.87	40	−1.5	12.05	38	13.1%	2.22 [-3.54, 7.98]
**Monika et al.**	2.98	5.26	65	1.65	4.56	61	15.7%	1.33 [-0.39, 3.05]
**Patel non-RCT**	20.4	11.4	20	0.5	14.5	20	11.2%	19.90 [11.82, 27.98]
**Patel RCT**	26.1	16.5	17	8.9	14.5	17	9.3%	17.20 [6.76, 27.64]
**Thangam et al.**	24.1	8.6361	20	−0.66	9.4673	15	12.8%	24.76 [18.65, 30.87]
**Total (95% CI)**	–		239	–		243	100.0%	12.03 [7.12, 16.93]

**Heterogeneity:** Tau^2^ = 39.08; Chi^2^ = 95.81, *df* = 7 (*p* < 0.00001); *I*^*2*^ = 93%.

**Test for overall effect:***Z* = 4.80 (*p* < 0.00001).

**Table 2(b):** Statistical Computations of the Meta-analysis of all the included RCTs and non-RCTs for the outcome of Diastolic Blood Pressure.

**Study ID**	**Yoga Nidra**			**Control**			**Weight**	**Mean Difference**
**First Author**	**Mean**	**SD**	**Sample Size (*****n*****)**	**Mean**	**SD**	**Sample Size (*****n*****)**	**in %**	**IV, Random, 95% CI**
**Anjana et al.**	4.2	1.52	31	1.81	1.99	34	15.6%	2.39 [1.53, 3.25]
**Deepa et al.**	14.0	4.86	15	5.46	3.16	15	13.5%	8.54 [5.61, 11.47]
**Devraj et al.**	12.8	15.8993	31	2.68	11.104	43	8.5%	10.12 [3.61, 16.63]
**Lijun Li et al.**	−1.05	6.86	40	−1.85	5.99	38	13.6%	0.80 [-2.05, 3.65]
**Monika et al.**	4.22	4.99	65	3.47	4.05	61	15.1%	0.75 [-0.83, 2.33]
**Patel non-RCT**	14.2	7.5	20	2.1	6.2	20	11.6%	12.10 [7.84, 16.36]
**Patel RCT**	15.2	8.1	17	4.2	5.9	17	10.9%	11.00 [6.24, 15.76]
**Thangam et al.**	9.4	6.128	20	−0.53	7.0782	15	11.3%	9.93 [5.45, 14.41]
**Total (95% CI)**	–		239	–		243	100.0%	6.32 [3.53, 9.12]

**Heterogeneity:** Tau^2^ = 12.88; Chi^2^ = 67.82, *df* = 7 (*p* < 0.00001); *I*^*2*^ = 90%.

**Test for overall effect:***Z* = 4.43 (*p* < 0.00001).

**Fig. 4(a):**
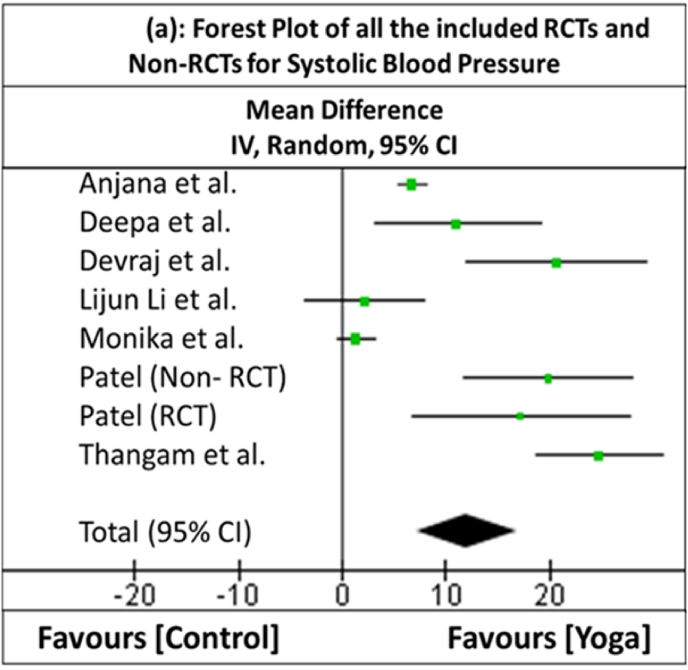
Forest plot of all the included RCTs and non-RCTs for systolic blood pressure.

**Fig. 4(b):**
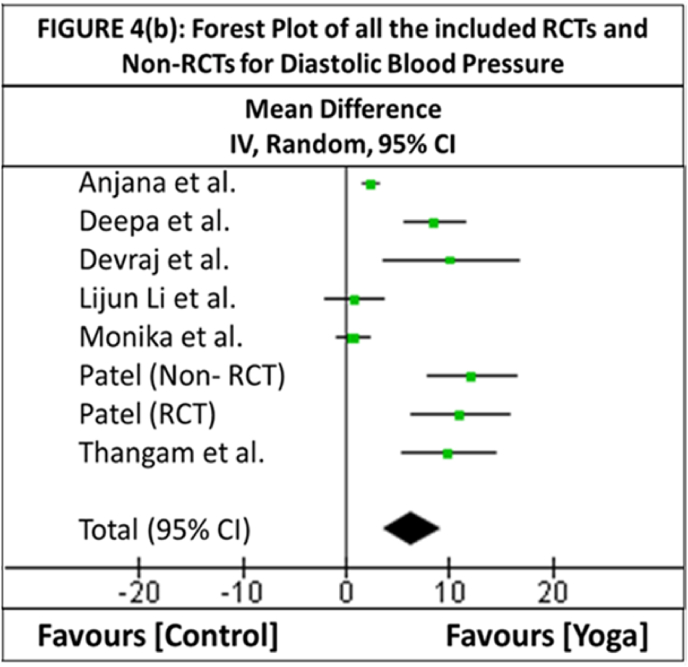
Forest plot of all the included RCTs and non-RCTs for diastolic blood pressure.

## 4 Discussion

Hypertension is a complex, multifactorial disease with numerous interrelated mechanisms contributing to its pathophysiology. The autonomic nervous system (ANS) and the central nervous system (CNS) play crucial roles in regulating BP. Sympathetic overactivity and increased sympathetic tone, coupled with decreased parasympathetic activity, contribute to hypertension [53]. Apart from the above, various other biomechanisms that may lead to the development of HTN include oxidative stress [54], insulin and leptin resistance [55,56], reduced baroreceptor sensitivity [57], poor circadian rhythm [58], mental stress, anxiety and depression [59,60], excessive anti-natriuretic factors such as aldosterone and endothelin-1 [61], diet and sodium intake [62], genetic factors [63], microbiome with gut dysbiosis [64], Renal dysfunction [65], and finally, vascular inflammation [66] driven by numerous factors such as oxidative stress, inflammation, and endothelial dysfunction, which also contribute to hypertension's development.

The impact of yoga on various physiological systems has been documented, and several mechanisms have been proposed regarding its beneficial role in hypertension. Yoga has a positive impact on the ANS because it successfully decreases SNS activity while simultaneously increasing PNS activity [67]. Yoga has been shown to reduce stress levels [68] and oxidative stress markers, such as malondialdehyde, while simultaneously increasing the presence of antioxidant enzymes, such as superoxide dismutase [69]. Yoga has also demonstrated the ability to enhance insulin sensitivity and mitigate leptin resistance [70,71], improve baroreceptor sensitivity and autonomic tone [72,73], and normalize the circadian rhythm by decreasing the levels of cortisol and elevating the levels of melatonin [74,75]. All of the above aid in the reduction of BP and a consequent enhancement in the individual's cardiovascular well-being.

There is ample evidence supporting how yoga alleviates symptoms of mental stress, anxiety, and depression by enhancing autonomic functions, activating neurohormonal processes, and suppressing sympathetic activity [73,76]. Incorporating yoga into one's lifestyle can even lead to improved dietary habits and a reduction in sodium intake [77,78], both of which have been associated with a decrease in BP. While the impact of yoga on genetics is yet to be fully explored, previous research indicates that these practices may have the ability to regulate gene expression and enhance cardiovascular health [[79], [80], [81]]. They also help improve the interactions of the gut-brain axis by fine-tuning the modulation of the gut microbiota through the CNS and ANS pathways [82,83]. This decreases inflammation, leading to potential indirect benefits for BP regulation. Studies also show that regular yoga practice can enhance renal function and mitigate proteinuria, thereby leading to a potential reduction in BP [84,85]. Yoga effectively decreases the presence of inflammatory markers, including C-reactive protein [86,87], leading to enhanced cardiovascular health and positively impacting BP.

Different yoga practices like asana, pranayama, meditation, YN, etc. have their own deep and intricate mechanisms in the body-mind complex, whose investigation is a vast topic in itself. The effect of YN on HTN can be attributed to its mechanism of primarily initiating a relaxation response, which reduces sympathetic activity and enhances parasympathetic activity. Thus, YN operates primarily on the brain by inducing relaxation throughout the neural system [19,88,89]. By lowering sympathetic overactivity and moderating heart rate, YN helps reduce peripheral vascular resistance and BP [21,89]. In addition, by addressing the mental health of hypertensive individuals, such as anxiety, depression, and stress, it has a secondary benefit in the reduction of BP [21]. Similar to meditation, YN might also help reduce vascular inflammation and, hence, have positive effects on BP [90]. Since YN does not involve physical postures or asanas and can be practiced in a comfortable supine position, it can prove to be an ideal practice for the elderly, who are at a higher risk of developing hypertension and may have difficulty exercising and following an active lifestyle [91]. Thus, YN can be a potential therapeutic modality for hypertension. However, there is a paucity of research on YN [21,88,89]; for example, the effect of YN on the underlying mechanisms of HTN can be explored, and further research is required to comprehensively study the potential mechanisms of YN on BP and hypertension.

Our results on YN are consistent with previous review studies [[92], [93], [94], [95]] assessing the effects of other yoga interventions on BP. They have reported favorable effects of yoga on hypertension, and the current evidence points to positive outcomes for YN as a therapeutic modality. However, we need to further explore the evidence and mechanisms so that the practices of yoga and YN in particular may be adapted more in clinics as well as introduced in the therapeutic guidelines for hypertension once substantial evidence is generated.

Also, it is to be noted that yoga comprised different practices in almost all of the studies. We should utilize the common mechanisms and goals behind these and try to minimize the specific techniques and approaches that vary widely depending upon the tradition, teacher, and individual practitioner [[17], [18], [19]]. Hence, there is a need to quantify the practices of YN to better understand its therapeutic potential [18,20]. YN has been shown to have a wide range of physical, mental, and emotional benefits, and it is important to understand how these benefits can be maximized in different contexts. For future studies, specific ways to standardize and quantify the practices should be utilized to better understand their therapeutic potential. For example, using specific YN protocols, standardized outcome measures, and consistent training and certification standards can help assess the effects of YN on different health outcomes more accurately and make evidence-based recommendations for its use in clinical settings.

The strengths of our study are that, to the best of our knowledge, this is the first meta-analysis to assess the effects of YN on BP. The limitations of the study are that the overall risk of bias for all five non-RCTs was reported to be serious, while the overall risk of bias for two out of three RCTs was reported to be high. While it is difficult to control for individual differences in intervention trials, bias due to confounding variables such as age, gender, diet, lifestyle, comorbidities, etc. can be minimized by following a robust RCT study design or by carefully matching participants. Research conducted among highly homogenous populations, such as military personnel, the elderly at old age homes, prison inmates, etc., might help to minimize heterogeneity and confounding as participants in these scenarios adhere to a common dietary regimen, circadian cycle, and lifestyle [96].

Studies in the current review lacked participant blinding, while assessor blinding was still implemented in some studies since there is a practical problem of blinding participants regarding the intervention provided in the case of yoga interventions. The authors propose that special scoring criteria and bias evaluation instruments such as the “Risk of Bias Justification Table” (RATIONALE) could be utilized and developed in order to improve the design and evaluation of behavioral intervention studies such as yoga [97]. Another limitation is that the studies differed in the treatments delivered to the subjects; apart from YN, some trial participants took antihypertensive medications while others did not. This resulted in the heterogeneity of our results.

To add to the methodological rigor and improve the strength of the evidence, future researchers should keep a few pointers in mind while conducting RCTs, such as having a well-defined pre-trial protocol, a statistical analysis plan, considering a homogeneous population with a large sample size, using standardized interventions, having active comparator control groups, using standardized and validated outcome measures, adhering to planned timepoints, conducting longer follow-ups, and using intention-to-treat analysis in line with the protocol [96]. Following appropriate reporting guidelines like the “Consolidated Standards of Reporting Trials” (CONSORT) [98], or the “CheckList stAndardising the Reporting of Interventions For Yoga” (CLARIFY) guidelines in case of Yoga based studies, is equally important for the meticulous and transparent dissemination of the research findings [99].

Our chosen topic of review had a limited availability of good-quality studies, which led to a relatively small number of studies being included. This may have resulted in a narrower scope of analysis and reduced statistical power, which could have affected the generalizability and validity of the review's conclusions. Due to the scarcity of good-quality studies, this review had to include studies with different designs, settings, and populations, which added to the heterogeneity, making it more challenging to draw meaningful conclusions from the pooled data. Furthermore, the lack of high-quality studies may have led to a potential for publication bias and an overestimation of the size of the effect of the intervention being studied. Lastly, a number of factors—including small sample sizes, methodological flaws, and brief follow-up periods—may have reduced the quality of the studies included in this review. These factors could have affected the accuracy and generalizability of the study's findings.

Despite these challenges, there are several reasons why there is a need to generate more interest in conducting trials on YN. YN provides distinct advantages for individuals with HTN, addressing the physical, mental, and neurological dimensions simultaneously. YN has the capacity to promote profound relaxation, diminish stress, and enhance parasympathetic activity, which is one of the mechanisms through which it reduces HTN. Additionally, its low-cost and non-invasive nature, along with its simplicity in terms of instructions, demonstration, accessibility, and adaptability, add to YN's potential to benefit a wide range of populations, including those who may not be able to perform physical exercise or yoga asana or pranayama.

As mentioned earlier, there is a growing body of research suggesting that YN itself may be an effective adjunct therapy for many health conditions. As such, there is a need to better understand how this practice can be tailored to different populations and conditions in order to maximize its therapeutic potential. Finally, there is a need to generate more interest in YN research in order to increase public awareness of the benefits of YN and promote its integration into mainstream healthcare settings. By conducting high-quality scientific studies on YN, further evidence can be generated to support its use in clinical practice and help to overcome some of the skepticism that still exists around complementary and alternative therapies.

## 5 Conclusion

A significant overall effect was observed in favor of YN for a sample size of 682 participants (239 experimental group vs. 243 controls), with an overall reduction as Mean Difference of 12.03 (7.12 ± 16.93) mm Hg for SBP and 6.32 (3.53 ± 9.12) mm Hg for DBP. The current review establishes a strong basis for YN to be used as an alternative and complementary treatment modality for managing hypertension, however, there is a dearth of research evidence available till date and further trials are needed to generalize these findings. With the growing use of internet, audio-visual and mobile based applications, YN might prove to be an attractive, cost effective, easily accessible, simple and safe treatment modality for the major worldwide health issue of Hypertension. Additional studies on YN should be conducted with a heightened understanding of the risk for bias and trial quality assessments while integrating additional tools and techniques for quantifying the psychological, neurological, endocrinological, immunological effects of YN on BP and hypertension. High-quality RCTs with rigorous methodology are needed to investigate standardized and validated YN programs as treatment and preventive modalities for hypertension, not only as a supplemental therapy but also as a primary intervention. Future studies should focus on larger sample sizes, longer follow-up periods, and more rigorous study designs to provide more robust evidence on the effectiveness of YN for hypertension management. This could include randomized controlled trials that compare the effects of YN to other therapies, as well as studies that investigate the underlying mechanisms of action.

## Sources of funding

None.

## Author contributions

NA: Conceptualization, Methodology, Investigation, Data Curation, Software, Formal Analysis, Writing – Original Draft. PB: Conceptualization, Methodology, Investigation, Data Curation, Formal Analysis, Visualization, Writing – Original Draft. MP: Conceptualization, Methodology, Validation, Writing – Review & Editing, Supervision. DS: Methodology, Investigation, Data Curation, Formal Analysis, Visualization, Writing – Original Draft. AK: Data Curation, Writing – Original Draft. AP1: Data Curation, Writing – Original Draft. AC: Investigation, Writing – Review & Editing. AP2: Conceptualization, Writing – Review & Editing.

## Ethical statement

Institutional ethics permissions were not required for carrying out this review.

## Data availability

The data utilized for this review can be made available by the authors upon reasonable request.

## Declaration of competing interest

The authors declare that they have no known competing financial interests or personal relationships that could have appeared to influence the work reported in this paper.

## References

[1] Whelton P.K., Carey R.M., Aronow W.S., Casey D.E., Collins K.J., Dennison Himmelfarb C. (2017). ACC/AHA/AAPA/ABC/ACPM/AGS/APhA/ASH/ASPC/NMA/PCNA guideline for the prevention, detection, evaluation, and management of high blood pressure in adults: a report of the American College of Cardiology/American Heart Association Task Force on Clinical Practice Guidelines. J Am Coll Cardiol.

[2] NCD Risk Factor Collaboration (NCD-RisC) (2021). Worldwide trends in hypertension prevalence and progress in treatment and control from 1990 to 2019: a pooled analysis of 1201 population-representative studies with 104 million participants. Lancet.

[3] Anchala R., Kannuri N.K., Pant H., Khan H., Franco O.H., Di Angelantonio E. (2014). Hypertension in India: a systematic review and meta-analysis of prevalence, awareness, and control of hypertension. J Hypertens.

[4] Arima H., Barzi F., Chalmers J. (2011). Mortality patterns in hypertension. J Hypertens.

[5] Murray C.J., Aravkin A.Y., Zheng P., Abbafati C., Abbas K.M., Abbasi-Kangevari M. (2020). Global burden of 87 risk factors in 204 countries and territories, 1990–2019: a systematic analysis for the Global Burden of Disease Study 2019. Lancet.

[6] Roth G.A., Mensah G.A., Johnson C.O., Addolorato G., Ammirati E., Baddour L.M. (2020). Global burden of cardiovascular diseases and risk factors, 1990–2019: update from the GBD 2019 study. J Am Coll Cardiol.

[7] Harrison D.G., Coffman T.M., Wilcox C.S. (2021). Pathophysiology of hypertension: the mosaic theory and beyond. Circ Res.

[8] Hagins M., Selfe T., Innes K. (2013). Effectiveness of yoga for hypertension: systematic review and meta-analysis. Evid Based Complement Alternat Med.

[9] Olowofela A.O., Isah A.O. (2017). A profile of adverse effects of antihypertensive medicines in a tertiary care clinic in Nigeria. Ann Afr Med.

[10] Gebreyohannes E.A., Bhagavathula A.S., Abebe T.B., Tefera Y.G., Abegaz T.M. (2019). Adverse effects and non-adherence to antihypertensive medications in university of gondar comprehensive specialized hospital. Clin Hypertens.

[11] Kretchy I.A., Owusu-Daaku F.T., Danquah S.A. (2014). Mental health in hypertension: assessing symptoms of anxiety, depression and stress on anti-hypertensive medication adherence. Int J Ment Health Syst.

[12] Jarari N., Rao N., Peela J.R., Ellafi K.A., Shakila S., Said A.R. (2015). A review on prescribing patterns of antihypertensive drugs. Clin Hypertens.

[13] Alefan Q., Huwari D., Alshogran O.Y., Jarrah M.I. (2019). Factors affecting hypertensive patients' compliance with healthy lifestyle. Patient Prefer Adherence.

[14] Cramer H., Haller H., Lauche R., Steckhan N., Michalsen A., Dobos G. (2014). A systematic review and meta-analysis of yoga for hypertension. Am J Hypertens.

[15] Posadzki P., Cramer H., Kuzdzal A., Lee M.S., Ernst E. (2014). Yoga for hypertension: a systematic review of randomized clinical trials. Compl Ther Med.

[16] Levine G.N., Lange R.A., Bairey‐Merz C.N., Davidson R.J., Jamerson K., Mehta P.K. (2017). Meditation and cardiovascular risk reduction: a scientific statement from the American Heart Association. J Am Heart Assoc.

[17] Saraswati S.S., Hiti J.K. (1984).

[18] Hoye S., Reddy S. (2016). Yoga-nidra and hypnosis. Int J Health Promot Educ.

[19] Aurobindo S. (2004).

[20] Musto S., Vallerand A.H. (2023). Exploring the uses of yoga nidra: an integrative review. J Nurs Scholarsh.

[21] Reboussin D.M., Allen N.B., Griswold M.E., Guallar E., Hong Y., Lackland D.T. (2018). Systematic review for the 2017 ACC/AHA/AAPA/ABC/ACPM/AGS/APhA/ASH/ASPC/NMA/PCNA guideline for the prevention, detection, evaluation, and management of high blood pressure in adults: a report of the American College of Cardiology/American Heart Association Task Force on Clinical Practice Guidelines. Hypertension.

[22] Ouzzani M., Hammady H., Fedorowicz Z., Elmagarmid A. (2016). Rayyan — a web and mobile app for systematic reviews. Syst Rev.

[23] Khandekar J.S., Vasavi V.L., Singh V.P., Samuel S.R., Sudhan S.G., Khandelwal B. (2021). Effect of yoga on blood pressure in prehypertension: a systematic review and meta-analysis. Sci World J.

[24] Nalbant G., Hassanein Z.M., Lewis S., Chattopadhyay K. (2022). Content, structure, and delivery characteristics of yoga interventions for managing hypertension: a systematic review and meta-analysis of randomized controlled trials. Front Public Health.

[25] Cramer H., Lauche R., Anheyer D., Pilkington K., de Manincor M., Dobos G. (2018). Yoga for anxiety: a systematic review and meta‐analysis of randomized controlled trials. Depress Anxiety.

[26] Curtis K., Weinrib A., Katz J. (2012). Systematic review of yoga for pregnant women: current status and future directions. Evid Based Complement Alternat Med.

[27] Innes K.E., Vincent H.K. (2007). The influence of yoga-based programs on risk profiles in adults with type 2 diabetes mellitus: a systematic review. Evid Based Complement Alternat Med.

[28] Yang K. (2007). A review of yoga programs for four leading risk factors of chronic diseases. Evid Based Complement Alternat Med.

[29] Park S.H., Han K.S. (2017). Blood pressure response to meditation and yoga: a systematic review and meta-analysis. J Alternative Compl Med.

[30] Kumar K. (2005). Effect of Yoga nidra on hypertension & other psychological co-relates. Yoga The Science.

[31] Vanitha A., Pandiaraja M., Maheshkumar K., Venkateswaran S.T. (2018). Effect of yoga nidra on resting cardiovascular parameters in polycystic ovarian syndrome women. Natl J Physiol Pharm Pharmacol.

[32] Patel C.H. (1973). Yoga and BIO-feedback in the management of hypertension. Lancet.

[33] Datey K.K., Deshmukh S.N., Dalvi C.P., Vinekar S.L., Datey K.K. (1969). Shavasan": a yogic exercise in the management of hypertension. Angiology.

[34] Bera T.K., Gore M.M., Oak J.P. (1998). Recovery from stress in two different postures and in Shavasana-A yogic relaxation posture. Indian J Physiol Pharmacol.

[35] Dvivedi J.Y., Dvivedi S.A., Mahajan K.K., Mittal S.U., Singhal A.N. (2008). Effect of'61-points relaxation technique'on stress parameters in premenstrual syndrome. Indian J Physiol Pharmacol.

[36] Dhungana R.R., Pedisic Z., Joshi S., Khanal M.K., Kalauni O.P., Shakya A. (2021 Mar 20). Effects of a health worker-led 3-month yoga intervention on blood pressure of hypertensive patients: a randomised controlled multicentre trial in the primary care setting. BMC Publ Health.

[37] Fetter C., Marques J.R., De Souza L.A., Dartora D.R., Eibel B., Boll L.F. (2020). Additional improvement of respiratory technique on vascular function in hypertensive postmenopausal women following yoga or stretching video classes: the YOGINI study. Front Physiol.

[38] Innes K.E., Selfe T.K. (2012). The effects of a gentle yoga program on sleep, mood, and blood pressure in older women with restless legs syndrome (RLS): a preliminary randomized controlled trial. Evid Based Complement Alternat Med.

[39] Pal A., Srivastava N., Tiwari S., Verma N.S., Narain V.S., Agrawal G.G., Natu S.M., Kumar K. (2011). Effect of yogic practices on lipid profile and body fat composition in patients of coronary artery disease. Compl Ther Med.

[40] Pal A., Srivastava N., Narain V.S., Agrawal G.G., Rani M. (2013). Effect of yogic intervention on the autonomic nervous system in the patients with coronary artery disease: a randomized controlled trial. East Mediterr Health J.

[41] Bhavanani A., Zeena S., Vithiyalakshmi L. (2012). Immediate cardiovascular effects of pranava relaxation in patients with hypertension and diabetes. Biomed Hum Kinet.

[42] Devi S., Kala S. (2015). Role of yoga-nidra and shirodhara on hypertensive patients. Int J Yoga Allied Sci.

[43] Cramer H., Thoms M.S., Anheyer D., Lauche R., Dobos G. (2016). Yoga in women with abdominal obesity—a randomized controlled trial. Dtsch Arztebl Int.

[44] Monika S.U., Ghildiyal A.R., Kala S.A., Srivastava N. (2012). Effect of Yoga Nidra on physiological variables in patients of menstrual disturbances of reproductive age group. Indian J Physiol Pharmacol.

[45] Li L., Shu W., Li Z., Liu Q., Wang H., Feng B., Ouyang Y.Q. (2019). Using yoga nidra recordings for pain management in patients undergoing colonoscopy. Pain Manag Nurs.

[46] Anjana K., Archana R., Mukkadan J.K. (2022). Effect of om chanting and yoga nidra on blood pressure and lipid profile in hypertension–A randomized controlled trial. J Ayurveda Integr Med.

[47] Deepa T., Sethu G., Thirrunavukkarasu N. (2012). Effect of yoga and meditation on mild to moderate essential hypertensives. J Clin Diagn Res.

[48] Patel C., North W.R. (1975). Randomised controlled trial of yoga and bio-feedback in management of hypertension. Lancet.

[49] Devraj J.P., Santosh Kumar B., Raja Sriswan M., Jagdish B., Priya B.S., Neelu S.B. (2021). Effect of yoganidra on blood pressure, Hs-CRP, and lipid profile of hypertensive subjects: a pilot study. Evid Based Complement Alternat Med.

[50] Patel C. (1975). 12-month follow-up of yoga and bio-feedback in the management of hypertension. Lancet.

[51] Thangam F.E., Bharathi A.D. (2019). Effect of yoga-nidra on blood pressure among elderly with hypertension residing at selected old age homes. Coimbatore. Int J Nurs Educ Res.

[52] Welch V.A., Petkovic J., Jull J., Hartling L., Klassen T., Kristjansson E., Higgins J.P.T., Thomas J., Chandler J., Cumpston M., Li T., Page M.J., Welch V.A. (2022). Cochrane Handbook for systematic reviews of interventions version 6.3 (updated february 2022).

[53] Fisher J.P., Young C.N., Fadel P.J. (2009). Central sympathetic overactivity: maladies and mechanisms. Auton Neurosci.

[54] Higashi Y. (2022). Roles of oxidative stress and inflammation in vascular endothelial dysfunction-related disease. Antioxidants.

[55] Reaven G.M. (1995). Pathophysiology of insulin resistance in human disease. Physiol Rev.

[56] Hall J.E., do Carmo J.M., da Silva A.A., Wang Z., Hall M.E. (2015). Obesity-induced hypertension: interaction of neurohumoral and renal mechanisms. Circ Res.

[57] Parati G., Esler M. (2012). The human sympathetic nervous system: its relevance in hypertension and heart failure. Eur Heart J.

[58] Makarem N., Alcántara C., Williams N., Bello N.A., Abdalla M. (2021). Effect of sleep disturbances on blood pressure. Hypertension.

[59] Liu M.Y., Li N., Li W.A., Khan H. (2017). Association between psychosocial stress and hypertension: a systematic review and meta-analysis. Neurol Res.

[60] Hamer M., Steptoe A. (2012). Cortisol responses to mental stress and incident hypertension in healthy men and women. J Clin Endocrinol Metab.

[61] Kostov K. (2021). The causal relationship between endothelin-1 and hypertension: focusing on endothelial dysfunction, arterial stiffness, vascular remodeling, and blood pressure regulation. Life.

[62] He F.J., MacGregor G.A. (2011). Salt reduction lowers cardiovascular risk: meta-analysis of outcome trials. Lancet.

[63] Levy D., Ehret G.B., Rice K., Verwoert G.C., Launer L.J., Dehghan A. (2009). Genome-wide association study of blood pressure and hypertension. Nat Genet.

[64] Maiuolo J., Carresi C., Gliozzi M., Mollace R., Scarano F., Scicchitano M. (2022). The contribution of gut microbiota and endothelial dysfunction in the development of arterial hypertension in animal models and in humans. Int J Mol Sci.

[65] Pugh D., Gallacher P.J., Dhaun N. (2019). Management of hypertension in chronic kidney disease. Drugs.

[66] Harrison D.G., Marvar P.J., Titze J.M. (2012). Vascular inflammatory cells in hypertension. Front Physiol.

[67] Pascoe M.C., Thompson D.R., Ski C.F. (2017). Yoga, mindfulness-based stress reduction and stress-related physiological measures: a meta-analysis. Psychoneuroendocrinology.

[68] Chiesa A., Serretti A. (2010). A systematic review of neurobiological and clinical features of mindfulness meditations. Psychol Med.

[69] Zope S.A., Zope R.A. (2013). Sudarshan kriya yoga: breathing for health. Int J Yoga.

[70] Chu P., Gotink R.A., Yeh G.Y., Goldie S.J., Hunink M.M. (2016). The effectiveness of yoga in modifying risk factors for cardiovascular disease and metabolic syndrome: a systematic review and meta-analysis of randomized controlled trials. Eur J Prev Cardiol.

[71] Aljasir B., Bryson M., Al-Shehri B. (2010). Yoga practice for the management of type II diabetes mellitus in adults: a systematic review. Evid Based Complement Alternat Med.

[72] Anasuya B., Deepak K.K., Jaryal A.K., Narang R. (2020). Effect of slow breathing on autonomic tone & baroreflex sensitivity in yoga practitioners. Indian J Med Res.

[73] Brown R.P., Gerbarg P.L. (2005). Sudarshan Kriya yogic breathing in the treatment of stress, anxiety, and depression: part I—neurophysiologic model. J Alternative Compl Med.

[74] Nagendra R.P., Maruthai N., Kutty B.M. (2012). Meditation and its regulatory role on sleep. Front Neurol.

[75] Mohan U.P., Kunjiappan S., Pichiah T., Babkiewicz E., Maszczyk P., Arunachalam S. (2023). Exploring the role of melatonin in meditation on cardiovascular health. Biointerface Res Appl Chem.

[76] Sengupta P. (2012). Health impacts of yoga and pranayama: a state-of-the-art review. Int J Prev Med.

[77] Kota V., Kumar S., Acharya S. (2023). Lifestyle modification and nutrition in preventing prehypertension and hypertension—narrative review. Int J Nutr Pharmacol Neurol Dis.

[78] Watts A.W., Rydell S.A., Eisenberg M.E., Laska M.N., Neumark-Sztainer D. (2018). Yoga's potential for promoting healthy eating and physical activity behaviors among young adults: a mixed-methods study. Int J Behav Nutr Phys Activ.

[79] Saatcioglu F. (2013). Regulation of gene expression by yoga, meditation and related practices: a review of recent studies. Asian J Psychiatr.

[80] Qu S., Olafsrud S.M., Meza-Zepeda L.A., Saatcioglu F. (2013). Rapid gene expression changes in peripheral blood lymphocytes upon practice of a comprehensive yoga program. PLoS One.

[81] Buric I., Farias M., Jong J., Mee C., Brazil I.A. (2017). What is the molecular signature of mind–body interventions? A systematic review of gene expression changes induced by meditation and related practices. Front Immunol.

[82] Ningthoujam D.S., Singh N., Mukherjee S. (2021). Possible roles of cyclic meditation in regulation of the gut-brain Axis. Front Psychol.

[83] Househam A.M., Peterson C.T., Mills P.J., Chopra D. (2017). The effects of stress and meditation on the immune system, human microbiota, and epigenetics. Adv Mind Body Med.

[84] Gordon L., McGrowder D.A., Pena Y.T., Cabrera E., Lawrence-Wright M.B. (2013). Effect of yoga exercise therapy on oxidative stress indicators with end-stage renal disease on hemodialysis. Int J Yoga.

[85] Pandey R.K., Arya T.V., Kumar A., Yadav A. (2017). Effects of 6 months yoga program on renal functions and quality of life in patients suffering from chronic kidney disease. Int J Yoga.

[86] Innes K.E., Bourguignon C., Taylor A.G. (2005). Risk indices associated with the insulin resistance syndrome, cardiovascular disease, and possible protection with yoga: a systematic review. J Am Board Fam Pract.

[87] Estevao C. (2022). The role of yoga in inflammatory markers. Brain Behav Immun Health.

[88] Parker S., Bharati S.V., Fernandez M. (2013). Defining yoga-nidra: traditional accounts, physiological research, and future directions. Int J Yoga Therap.

[89] Pandi-Perumal S.R., Spence D.W., Srivastava N., Kanchibhotla D., Kumar K., Sharma G.S. (2022). The origin and clinical relevance of yoga nidra. Sleep Vigil.

[90] Amarasekera A.T., Chang D. (2019). Buddhist meditation for vascular function: a narrative review. Integr Med Res.

[91] Bhardwaj P., Pathania N., Pathania M., Rathaur V.K. (2021). Evidence-based yoga and ayurveda lifestyle practices for the geriatric population during Coronavirus Disease 2019 pandemic: a narrative. J Prim Care Spec.

[92] Cramer H., Lauche R., Haller H., Steckhan N., Michalsen A., Dobos G. (2014). Effects of yoga on cardiovascular disease risk factors: a systematic review and meta-analysis. Int J Cardiol.

[93] Wang J., Xiong X., Liu W. (2013). Yoga for essential hypertension: a systematic review. PLoS One.

[94] Deolindo C.S., Ribeiro M.W., Aratanha M.A., Afonso R.F., Irrmischer M., Kozasa E.H. (2020). A critical analysis on characterizing the meditation experience through the electroencephalogram. Front Syst Neurosci.

[95] Tyagi A., Cohen M. (2016). Yoga and heart rate variability: a comprehensive review of the literature. Int J Yoga.

[96] Gupta S., Dhawan A. (2022). Methodological issues in conducting yoga-and meditation-based research: a narrative review and research implications. J Ayurveda Integr Med.

[97] de Bruin M., McCambridge J., Prins J.M. (2015). Reducing the risk of bias in health behaviour change trials: improving trial design, reporting or bias assessment criteria? A review and case study. Psychol Health.

[98] Schulz K.F., Altman D.G., Moher D., CONSORT Group (2010). CONSORT 2010 Statement: updated guidelines for reporting parallel group randomised trials. BMJ.

[99] Moonaz S., Nault D., Cramer H., Ward L. (2021). Clarify 2021: explanation and elaboration of the Delphi-based guidelines for the reporting of yoga research. BMJ Open.

